# First-in-human EphA2-targeting PET imaging with the bicyclic radiotracer [^68^Ga]Ga-BCY18469 in pancreatic cancer patients: biodistribution, dosimetry and initial findings

**DOI:** 10.1007/s00259-026-07985-1

**Published:** 2026-06-10

**Authors:** Ann-Christin Eder, Christoph-Ferdinand Wielenberg, Mohamed Aymen Omrane, Aikaterini Klotsotyra, Katia Brüggemann, Mohamed El Fakiri, Heiko Becker, Michael Quante, Michael Mix, Matthias Eder, Philipp T. Meyer, Martin T. Freitag

**Affiliations:** 1 Department of Nuclear Medicine, University Medical Center Freiburg, Faculty of Medicine, University of Freiburg, Hugstetter Str. 55, 79106 Freiburg, Germany; 2 Division of Radiopharmaceutical Development, German Cancer Consortium (DKTK), partner site Freiburg, 79106, Freiburg, Germany and German Cancer Research Center, 69120 Heidelberg, Germany; 3 Department of Hematology, Oncology and Stem Cell Transplantation, Faculty of Medicine, University of Freiburg, University of Freiburg, Freiburg, Germany; 4 Department of Medicine II, Gastroenterology, Hepatology, Endocrinology and Infectious Diseases, Faculty of Medicine, Medical Center-University of Freiburg, Freiburg, Germany

**Keywords:** EphA2, Bicyclic peptide, PET, Tumor targeting, Pan-cancer agent, PDAC

## Abstract

**Purpose:**

Erythropoietin-producing hepatocellular receptor A2 (EphA2) is overexpressed in various malignancies, including pancreatic ductal adenocarcinoma (PDAC), in which it correlates with poor prognosis. Although EphA2 is considered a promising target receptor for theranostic applications, suitable radiotracers for clinical imaging have been lacking. This study reports first clinical experiences with [^68^Ga]Ga-BCY18469, a bicyclic peptide radiotracer for EphA2-targeted PET imaging.

**Methods:**

Seven patients with histologically confirmed PDAC (5 after chemotherapy, 2 at initial staging) underwent PET/CT imaging. Four were scanned at 15, 30, 45, 60 and 180 min. and three at 45 min. after injection of [^68^Ga]Ga-BCY18469. Dosimetry calculations were performed based on organ-specific time-activity curves from whole-body PET acquisitions. Imaging findings were compared with contrast-enhanced CT or MRI (interval: 9–50 days).

**Results:**

No adverse events were observed. The kidneys received the highest absorbed dose (0.31 ± 0.02 mGy/MBq), while the effective dose was 0.017 ± 0.002 mSv/MBq. [^68^Ga]Ga-BCY18469 demonstrated rapid tumor uptake at 15 min. post-injection with predominantly renal excretion. Of 45 total lesions EphA2-PET detected 36 lesions with tracer uptake suspicious for metastasis. 11 of 45 lesions were detected only on EphA2-PET, whereas 9 of 45 lesions were detected only on CT and/or MRI. The tracer identified 13 liver metastases (SUV_max_ 6.9 ± 3.4) and 13 lymph node metastases (SUV_max_ 5.0 ± 1.1), among other findings.

**Conclusions:**

Our initial clinical experiences demonstrate that [^68^Ga]Ga-BCY18469 enables safe, rapid, and high-contrast visualization of EphA2-expressing PDAC lesions. These results strongly support further investigation of [^68^Ga]Ga-BCY18469 as a diagnostic tool for EphA2-positive malignancies.

**Supplementary Information:**

The online version contains supplementary material available at https://doi.org/10.1007/s00259-026-07985-1.

## Introduction

The receptor tyrosine kinase Erythropoietin-producing hepatocellular receptor A2 (EphA2) plays a crucial role in cellular signaling pathways that regulate tissue boundaries, angiogenesis, and cell positioning during development [1, 2]. In adult tissues, physiological EphA2 expression remains minimal, yet increased expression has emerged as a hallmark of multiple malignancies [3]. Elevated EphA2 levels are characteristic of various solid tumors, including pancreatic ductal adenocarcinoma (PDAC), breast, colorectal, prostate, and lung carcinomas [4–9]. In PDAC, elevated levels of EphA2 are particularly associated with aggressive phenotypes, enhanced metastatic potential, and adverse clinical outcomes [10, 11].

This differential expression pattern between malignant and normal tissues has prompted extensive investigation of EphA2 as a diagnostic and therapeutic target. New diagnostic and therapeutic approaches seem interesting for diseases with a poor prognosis, such as PDAC [12]. From a diagnostic perspective, inaccurate initial staging can result in futile surgery [13]. Furthermore, EphA2-targeted theranostic approaches offer interesting opportunities, given the limited treatment options for PDAC [14].

Previous approaches targeting EphA2 investigated the applicability of kinase inhibitors and immunoconjugates [15]. The antibody-drug conjugate MEDI547, for instance, exhibited promise in preclinical models but failed to demonstrate clinical benefit in early-phase trials [16, 17]. Recently, novel constructs such as bicycle toxin conjugates have advanced to clinical testing, with BT5528 currently under evaluation [18, 19].

For molecular imaging, radiolabeled antibodies targeting EphA2 have already provided proof-of-concept validation. Studies employing ^89^Zr-labeled EphA2-targeting antibodies have achieved tumor visualization in preclinical models [20]. However, clinical translation has revealed significant limitations, particularly inadequate tumor penetration, which precludes diagnostic utility [21]. Furthermore, the fundamental necessity for delayed imaging and the high radiation burden associated with ^89^Zr represent additional drawbacks [22]. More recently, cyclic peptides have been explored as alternative scaffolds for EphA2-targeted radionuclide imaging. These include the bicyclic peptide [^18^F]AlF-ETN developed from the Bicycle^®^ peptides originating from the BCY6088 scaffold and the monocyclic peptide [^177^Lu]Lu-DOTA-CEMJ4, both of which have demonstrated promising results in preclinical studies [23–25].

Constrained peptide scaffolds offer significant advantages for the development of radiopharmaceuticals in this context. Bicyclic peptides achieve antibody-like affinities through their conformationally restricted architecture while maintaining low molecular weights (1.5-3 kDa) compatible with rapid tissue distribution and efficient renal elimination [26–28]. We recently developed BCY18469, a bicyclic peptide identified through systematic phage display screening and subsequent medicinal chemistry optimization [29]. This compound demonstrates high-affinity binding to human EphA2 (KD = 1.93 nM) and high tumor accumulation in EphA2-expressing mice xenografts (e.g. 19.5% ID/g at 1 h in HT1080 xenografts) with minimal retention in non-target tissues, yielding high imaging contrasts at early time points [29].

Here, we report first clinical experience using [^68^Ga]Ga-BCY18469 as a PET imaging agent for EphA2-expression in PDAC. We provide first-in-human data on safety, radiation dosimetry and biodistribution kinetics, and preliminary diagnostic performance of the radiopharmaceutical in patients with PDAC.

## Methods

### Patients

This retrospective analysis was conducted in accordance with the Declaration of Helsinki in its current form. The study was approved by the local ethics committee of Freiburg, Germany (25-1444-S1-retro). The radiopharmaceutical was administered under § 13(2b) of the German Medicinal Products Act (Arzneimittelgesetz, AMG) permitting individualized radiopharmaceutical preparation for selected patients. All patients received comprehensive information regarding the investigational nature of this imaging procedure and provided written informed consent prior to participation. Detailed patient characteristics are summarized in Table 1. Seven patients (4 of 7 female; mean age 67.4 ± 8.1 years) with histologically confirmed PDAC (5 patients after first- or second-line chemotherapy and 2 at the timepoint of initial staging) underwent PET/CT after injection of [^68^Ga]Ga-BCY18469 (EphA2-PET/CT). Contrast-enhanced CT imaging and/or contrast-enhanced MRI was available for comparison in all patients (median: 34 days; range 9–50 days). These examinations covered the anatomical regions evaluated on PET/CT, ensuring that all lesions assessed on PET/CT were within the field of view of the corresponding CT/MRI examinations.

**Table 1:** Patient characteristics

Patient	Age (years)	Gender	Weight (kg)	MBq	Histology	Type of histology	Surgery	Chemotherapy
1	69	f	53	146	Ductal Adenocarcinoma of the pancreatic head, G2	CT-guided pancreas biopsy	-	After second line chemotherapy
2	68	f	60	146	Ductal Adenocarcinoma of the pancreatic tail, G2	EUS-guided pancreas biopsy	-	After second line chemotherapy
3	61	f	63	150	Ductal Adenocarcinoma of the pancreatic tail, G3	EUS-guided pancreas biopsy	Received pancreatic head resection due to chronic mass-forming pancreatitis*	After second line chemotherapy
4	53	m	68	113	Ductal Adenocarcinoma of the pancreatic tail, G3	Liver biopsy	-	After second line chemotherapy
5	77	m	70	236	Ductal Adenocarcinoma of the pancreatic tail, G2	Liver biopsy	-	After first-line chemotherapy
6	78	m	60	224	Ductal Adenocarcinoma of the pancreatic head, G3	EUS-guided pancreas biopsy	-	Initial diagnosis
7	66	f	52	187	Ductal Adenocarcinoma of the pancreatic corpus, G3	EUS-guided pancreas biopsy	-	Initial diagnosis

* Pancreatic head resection was performed in August 2020 for chronic mass-forming pancreatitis, with incidental detection of a small, clinically irrelevant neuroendocrine tumor (size 0.4 cm); four years later, pancreatic ductal adenocarcinoma of the pancreatic tail was diagnosed by EUS-guided biopsy

### Radiopharmaceutical preparation of [^68^Ga]Ga-BCY18469

Prior to clinical translation, preclinical toxicological analysis of [^68^Ga]Ga-BCY18469 was available in accordance with ICH M (3) for microdosing pharmaceuticals to determine the no adverse effect level (NOAEL) of the peptide.

BCY18469 incorporates DOTA as a bifunctional chelator as previously described [29]; the structural formula is provided therein. The synthesis of the radiopharmaceutical [^68^Ga]Ga-BCY18469 was performed following stringent Good Manufacturing Practice (GMP) standards to guarantee consistent product quality, safety, and reproducibility. Production utilized an automated ModularLab PharmTracer synthesis system (Eckert & Ziegler) fitted with disposable sterile cassettes.

Gallium-68 in the form of [^68^Ga]GaCl₃ was obtained through elution from a commercial ^68^Ge/^68^Ga generator (GalliAd^®^, IRE Elit) using 0.1 M hydrochloric acid and delivered directly to the reaction vessel. This vessel contained a pre-prepared mixture of BCY18469 peptide, sodium acetate/HCl buffer (pH 4.5), SCX elution solution, ethanol and ascorbic acid solution as antioxidant.

After heating at 95 °C, the reaction mixture was diluted using 0.9% sodium chloride solution, purified using a C18 cartridge, effectively separating the radiolabeled compound from free gallium-68 and reaction by-products and sterile filtered through a 0.22 μm membrane into a sterile container. Post-filtration membrane integrity testing confirmed successful sterilization. Quality control release testing included endotoxin, sterility, radio-HPLC, UV-HPLC, GC and TLC. Radiochemical purity exceeded 99% and chemical purity exceeded 95% as confirmed by UV-HPLC, meeting all required specifications for clinical use.

### PET/CT acquisitions

Whole-body PET scans were acquired after 15, 30, 45, 60 min and 180 min after injection of 172 MBq ± 42 MBq [^68^Ga]Ga-BCY18469 from mid‐thigh to vertex, with four patients scanned at all time points and three patients only scanned at 45 min after injection. PET/CT scans were performed on a Siemens VISION digital PET/CT using time of flight capability, PET reconstruction parameters were 4 iterations, 5 subsets, Gaussian filtering, zoom 1.2 and a full-width-half-maximum of 4 mm. A low-dose CT-scan was performed for attenuation correction and anatomic correlation (120 kVp, 30 mAs, CareDose and CarekV enabled). A scan speed of 1.1 mm/s was used for the first four timepoints (15, 30, 45 and 60 min), and a scan speed of 0.6 mm/s was used for the late imaging timepoint (180 min). Patients were monitored for adverse events throughout the imaging procedure and explicitly asked to report any symptoms.

### Image analysis and dosimetry

Images were reviewed by a senior nuclear medicine physician and radiologist (MTF, 15 years of experience in oncologic imaging) and a resident nuclear medicine physician (CFW, 4 years of experience in oncologic imaging), using Syngo.Via (Siemens Healthineers, Erlangen, Germany). The time point selected for comparison of PET images with diagnostic CT/MRI scans was 45 min after injection for all patients. As only the primary tumour or a liver metastasis in this patient cohort had been histologically confirmed, metastatic (palliative) situation was defined by expert consensus (tumor board members) as is common practice and in accordance with the German national S3 guideline on exocrine pancreatic carcinoma [30]. These lesions were then included in further analyses. If available, subsequent imaging was used to assess whether suspected metastatic lesions could be confirmed. Lesions were confirmed as metastatic if they demonstrated progressive growth on follow-up imaging or, in the case of osseous lesions, increasing sclerotic changes under therapy.

For the assessment of standardized uptake value (SUV) in normal organs, representative volumes of interest (VOIs) were placed. To calculate maximum and mean SUV (SUVmax and SUVmean) in tumors, VOIs were drawn and a 40% isocontour of the SUVmax was calculated. For dosimetric assessment, relevant organs were segmented using MIM Software (v 7.4.3, MIM Software Inc, Cleveland, OH, USA) to determine organ activity concentrations and extract time activity data, tumor-to-background ratios and activity residence times.

Time-activity curves (TACs) for all organs of interest were generated using monoexponential fits in Microsoft Excel (Microsoft Corp., Redmond, WA, USA), with the exception of the urinary bladder and the kidneys.

For the urinary bladder, a dynamic bladder model [31] was applied to estimate the radiation dose absorbed by the urinary bladder wall, taking into account the decay of the radiopharmaceutical activity within the bladder. The model simulates the changes in bladder volume due to continuous filling with urine and subsequent voiding. In this case the bladder uptake was modelled using the excretion rate of the kidney to account for bladder filling and voiding after the PET scan (1.25 h post-injection) and then after every 3 h. Renal activity was modelled during the first 15 min using fast monoexponential kinetics, resulting in excretion of the measured activity into the bladder 15 min after injection. A monoexponential curve was fitted for the measured activity at later time points with a slower kinetic.

These curve equations were applied to calculate the time-integrated activity (TIA) and time-integrated activity coefficient (TIAC) for each organ. The TIAC values served as input for IDAC-Dose Software (IDAC-Dose 2.1), which is based on the specific absorbed fractions from the ICRP Publication 133 for the absorbed dose calculation in each organ of interest [32, 33].

## Results

### Safety and dosimetry

**Fig. 1:**
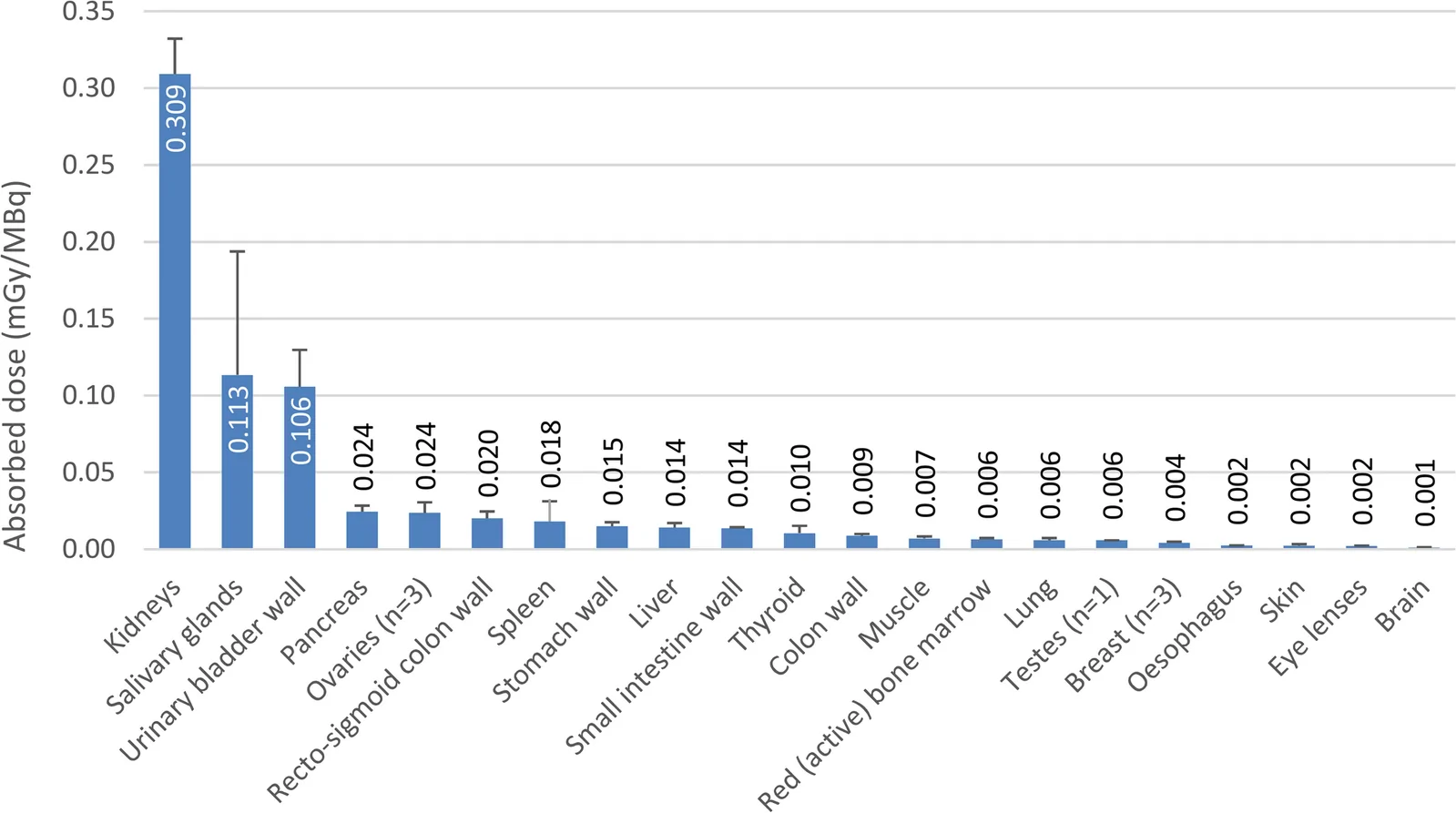
- Mean organ absorbed doses with standard deviation for 4 patients (3 female and 1 male)

No adverse events or clinically noticeable pharmacologic effects were observed in any of the patients. To evaluate the radiation safety profile of [^68^Ga]Ga-BCY18469, absorbed dose values were calculated. In all four patients the dose limiting organs were the kidneys (*0.31 ± 0.02* mGy/MBq), followed by the salivary glands (*0.11 ± 0.08* mGy/MBq) and the urinary bladder wall (*0.11 ± 0.02* mGy/MBq). The mean absorbed doses for all organs calculated using the ICRP 133 phantoms and patient specific organ masses are shown in Fig. 1 and supplementary Table 1. The effective dose applying tissue weighting factors according to ICRP Publication 103 is *0.017 ± 0.002 mSv/MBq*, resulting in 2.55 ± 0.3 mSv per 150 MBq [^68^Ga]Ga-BCY18469 [34].

### Imaging findings

**Fig. 2:**
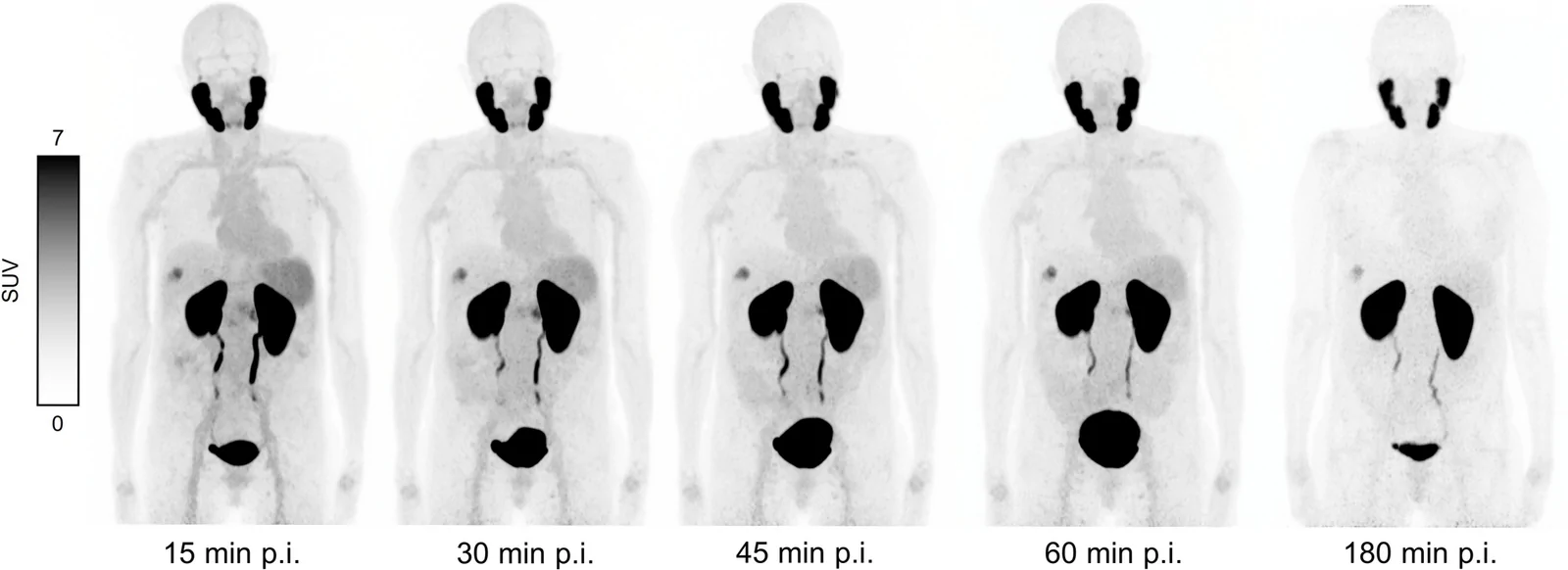
Maximum intensity projections (MIPs) at five timepoints. MIPs acquired 15, 30, 45, 60 and 180 min after injection of [^68^Ga]Ga-BCY18469 in a patient (patient 3 in Table 2) with pancreatic ductal adenocarcinoma and singular liver metastasis

**Fig. 3:**
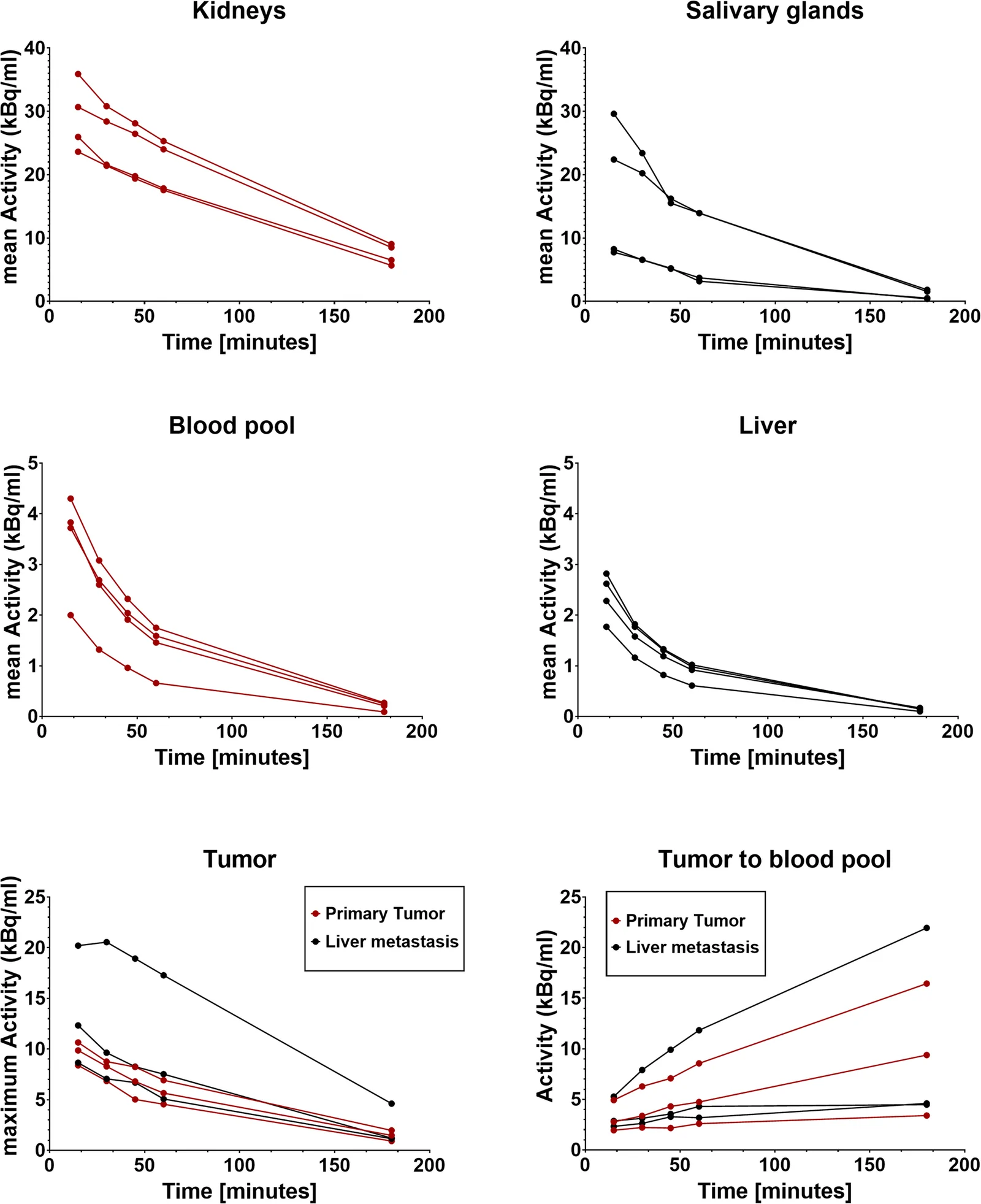
Time–activity curves (TACs) of four patients were obtained for the kidneys, salivary glands, blood pool, liver, tumor, and the tumor-to-blood pool ratio at 15, 30, 45, 60, and 180 min after injection of [^68^Ga]Ga-BCY18469. For each patient, the activity value measured at the respective time point was plotted individually. For physiological organ uptake, mean activity concentrations were used, whereas maximum activity concentrations were used for tumor uptake. TACs of the kidneys and salivary glands showed high activity concentrations with a gradual decrease over all time points. Blood pool and liver showed lower activity concentrations with a rapid decrease over time. Tumor TACs also demonstrated a decrease over the 180-minute measurement period, while the tumor-to–blood pool ratio increased over time. As a primary tumor was available in only three of the four patients, the liver metastasis with the highest uptake, when present, was additionally analyzed

**Fig. 4:**
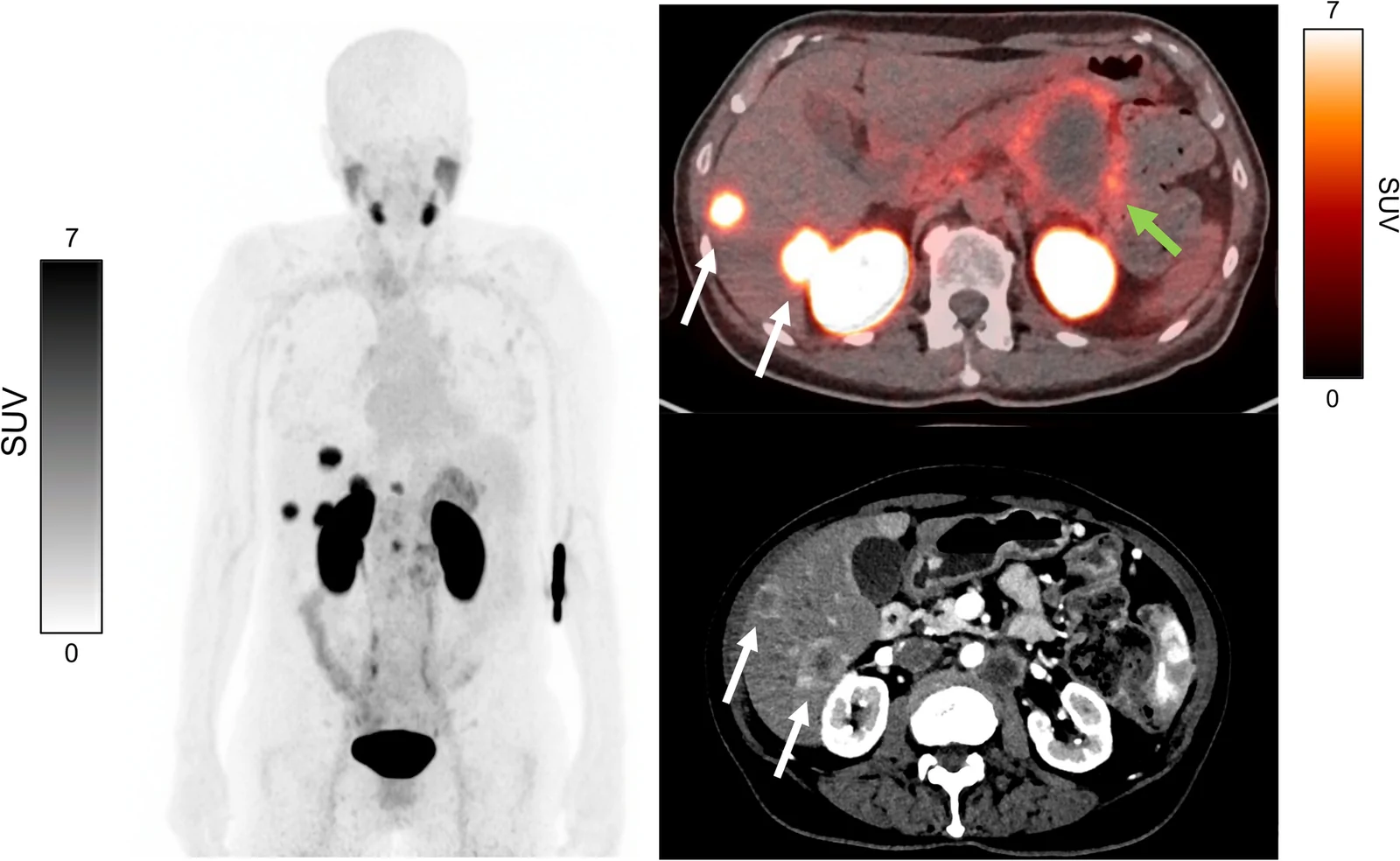
Comparison of standardized uptake values (SUV) between primary, different kinds of metastasis, physiologic uptake in the liver and blood pool. The maximum SUV (SUVmax) is shown in green, and the mean SUV (SUVmean) is shown in black

Images were considered diagnostic in all cases. The radiotracer demonstrated significant uptake in tumor lesions already at 15 min post injection (p.i.), followed by a decrease over the subsequent time points (for a representative patient, see Fig. 2; for TACs see Fig. 3). Predominant excretion occurred via the kidneys, as reflected by the high absorbed renal dose. The salivary glands also showed high uptake with a decrease over all time points (Fig. 3), with notable interindividual variability among patients. Uptake in the blood pool (SUVmean 2.5 ± 1.3 at 45 min p.i.) and liver (SUVmean 0.9 ± 0.3 at 45 min p.i.) was favorably low and decreased rapidly over time (see Figs. 3 and 4). Importantly, the tumor-to-blood pool ratio increased consistently across all five time points (see Fig. 3).

**Table 2:** Comparison of PET/CT 45 min after administration of [^68^Ga]Ga-BCY18469 to conventional staging. Patients 1–5 are pretreated, patients 6 and 7 untreated at initial diagnosis

Finding	Patient 1	Patient 2	Patient 3	Patient 4	Patient 5	Patient 6	Patient 7
Lesions detected by CT/MRI and PET	1	8	2	6	6	1	1
Discordant lesions not detected by PET	2^∗^	0	0	0	7^∗∗^	0	0
Lesions detected by PET only	0	0	1	0	1	2	7
PET-positive lesions confirmed by either biopsy or follow-up	1^‡^	1^‡^	3	6	1^‡^	3	8
Lesion SUV_max_ range	4.0	5.0–13.7	3.3–5.9	3.7–6.5	3.0–5.1	3.9–5.3	3.6–7.8
Time between PET/CT and conventional imaging (days)	21	9	50	34	46	12	34

* Lung lesions with a maximum axial diameter of 2.2 × 1.6 cm, pretreated patient

^**^ Cystic configurated liver lesions with a maximum axial diameter of 5.6 × 4.4 cm, pretreated patient

^‡^ patient did not undergo any further examination after the PET/CT

**Fig. 5:**
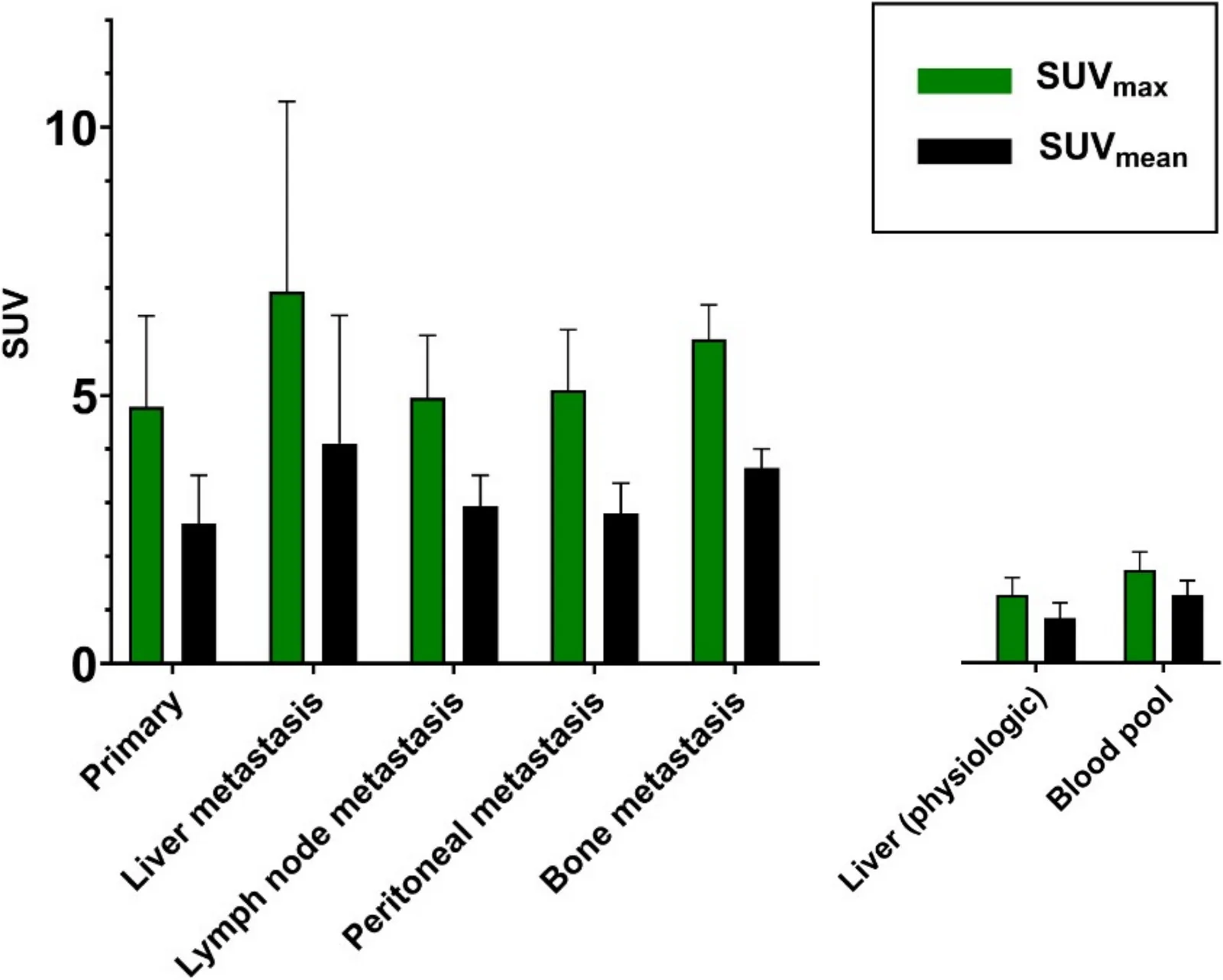
Patient (patient 2 in Table 2) with lymphonodal and hepatic metastasized pancreatic ductal adenocarcinoma. Maximum intensity projection (on the left site) acquired 45 min after injection of [^68^Ga]Ga-BCY18469. Fused axial PET/CT images (on the upper right site) 45 min after injection of [^68^Ga]Ga-BCY18469 showing pancreatic tumor mass (green arrow) and hepatic metastases (white arrows). Corresponding liver lesions (white arrows) are depicted on the CT scan obtained during the arterial contrast phase (on the lower right site). Note that minor differences in the anatomical depiction of the liver and primary tumor between the contrast-enhanced CT and the PET/CT fusion images are attributable to differences in slice thickness and respiratory phase at the time of acquisition (nine days interval)

Of 45 total lesions identified by any modality, PET detected 36 lesions (25 concordant with CT/MRI, 11 PET-only), while 9 lesions were detected only by CT/MRI (see Table 2 for details). Figure 5 shows an example image of a patient with liver and lymph node metastases. EphA2-PET detected 13 liver metastases (SUVmax 6.9 ± 3.4), 13 lymph node metastases (SUVmax 5.0 ± 1.1), 2 bone metastases (SUVmax 6.1 ± 0.5), and 2 peritoneal metastases (SUVmax 5.1 ± 0.8), see Fig. 4. Primary tumor uptake was observed in 6 of 7 patients, albeit with lower uptake compared to liver metastases (SUVmax 4.8 ± 1.6), see Fig. 4. In the patient in whom no uptake of the primary tumor was observed, there was also no morphological correlate in conventional imaging.

In total, 11 of 45 lesions were detected on EphA2 PET but not identified on conventional imaging (CT and/or MRI). These lesions comprised 2 peritoneal lesions, 8 lymph node metastases, and a liver metastasis. Noteworthy, both patients without prior treatment showed suspected lesions on EphA2 PET that were not detected on CT or MRI. The suspicious lesions not detected by EphA2-PET comprised 2 lung metastases with a maximum axial diameter of 2.2 × 1.6 cm in one patient and 7 cystically configured liver metastases with a maximum axial diameter of 5.6 × 4.4 cm in another patient.

Of the 45 lesions identified as suspicious, *5 lesions represented histologically confirmed primary tumors (pancreatic biopsy in patients 1–3*,* 6 and 7)*,* and 2 additional lesions (liver metastases in patients 4 and 5) were histologically confirmed via liver biopsy.* Furthermore, 16 lesions in four patients (8 lymph node metastases, 5 liver metastases, 2 bone metastases, and 1 peritoneal metastasis) were considered confirmed metastases based on subsequent imaging. The remaining 22 lesions in three patients (patients 1, 2, and 5 in Tables 1 and 2) could not be further assessed, as no follow-up imaging was available.

**Fig. 6:**
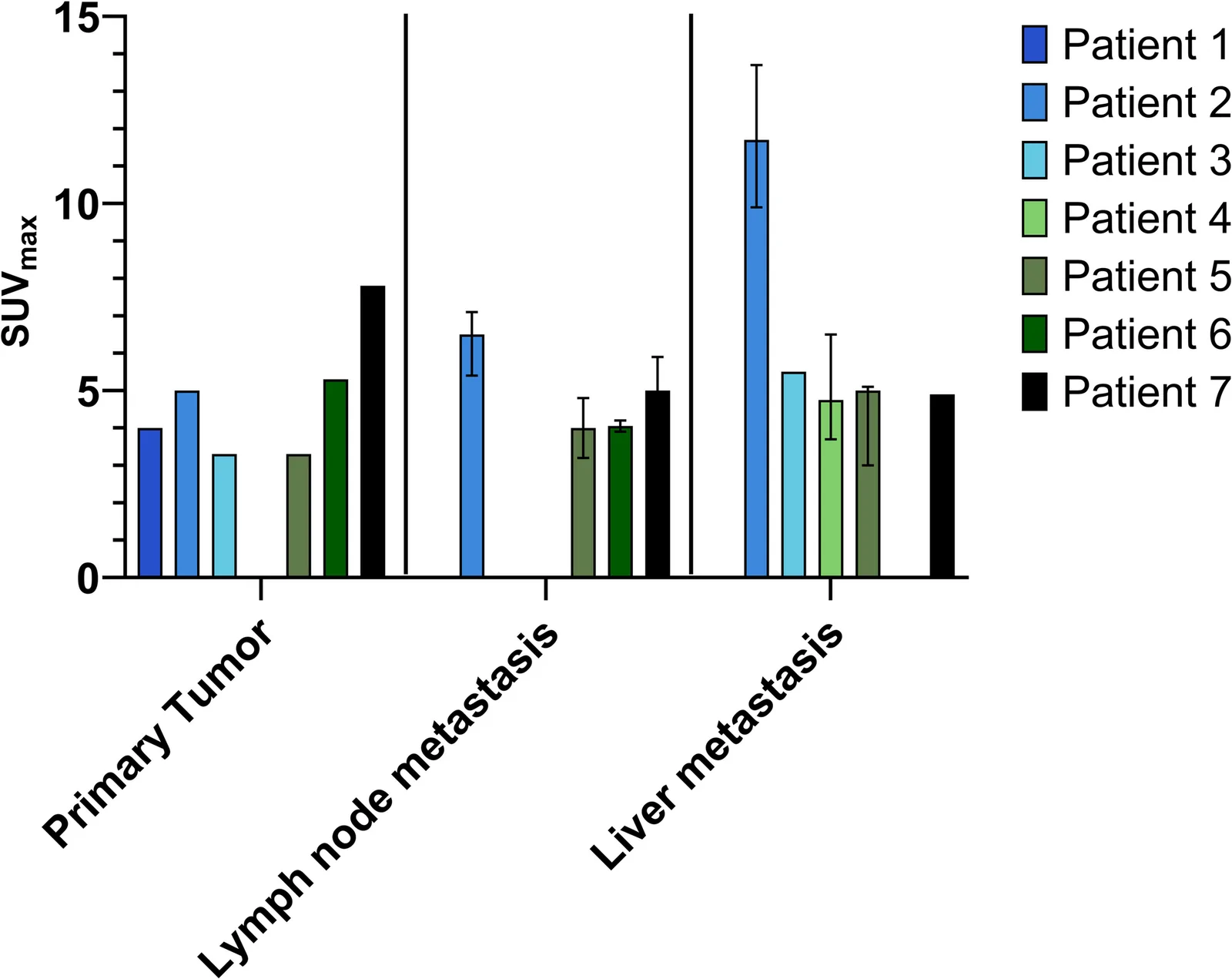
Patient-wise comparison of maximum standardized uptake values (SUV_max_) for primary tumor, lymph node metastasis, and liver metastasis (where applicable median and range are shown). Patients without prior therapy (patients 6 and 7) tended to show higher uptake in the primary tumor than patients with prior therapies. Interindividual variability in liver metastasis uptake was also observed. Other metastatic sites (bone and peritoneal) were not considered due to the small number of cases (*n* = 4 in 3 patients)

The two patients without prior therapy (patients 6 and 7 in Tables 1 and 2) tended to show higher uptake in the primary tumor than patients with prior therapies (patients 1–5 in Tables 1 and 2; Fig. 6). Compared with the primary tumor, lower uptake was observed in metastatic lesions in patients without prior therapy (Fig. 6). In addition, marked interindividual variability in liver metastasis uptake was observed (Fig. 6).

Potential clinical benefit from PET imaging was demonstrated in 2 of 7 patients with newly diagnosed PDAC (patients 6 and 7 in Tables 1 and 2): in the first patient, initially considered borderline resectable, PET imaging revealed tracer uptake in retroperitoneal lymph nodes, which showed progression on subsequent imaging. In the second patient, PET identified a small liver lesion not evident on CT imaging performed one month prior and confirmed by subsequent follow-up imaging as growing liver metastasis.

## Discussion

In this evaluation, we deliver first-in-human data on EphA2-specific PET imaging and show that [^68^Ga]Ga-BCY18469 enables rapid and high-contrast visualization of EphA2‐expressing tumor lesions in patients with PDAC. The tracer showed favorable pharmacokinetics with fast tumor uptake, rapid background clearance, and predominantly renal excretion. Particularly noteworthy was the low physiological liver uptake, which is considered to be highly advantageous for detecting hepatic metastases, the most frequent metastatic pattern in PDAC.

Quantitative dosimetry showed a favorable radiation safety profile. The kidneys received the highest absorbed dose (*0.31 ± 0.02* mGy/MBq) with renal excretion as the dominant clearance route. Similar organ-dose patterns have been reported for other ^68^Ga-labeled tracers. For instance, the kidney doses for [^68^Ga]Ga-PSMA-11 and [^68^Ga]Ga-DOTATATE were 0.24 ± 0.04 mGy/MBq [35] and 0.14 ± 0.05 mGy/MBq [36], respectively.

Similar to other peptide-based radiopharmaceuticals, such as PSMA-targeted tracers, the salivary glands showed moderate absorbed doses (*0.11 ± 0.08* mGy/MBq). This could reflect either EphA2 expression in secretory epithelial structures, or alternatively, receptor-independent retention due to non-specific tracer uptake. No organs demonstrated tracer accumulation that would be expected to impose dose-limiting toxicity in future therapeutic applications utilizing the same binding scaffold with therapeutic radionuclides. This assumption has to be confirmed by a separate dosimetry with the therapeutic compound in the future.

To our knowledge, other clinical data on EphA2-targeted PET imaging are limited. Previous clinical evaluation of the antibody-based approach with ^89^Zr-DS-8895a demonstrated insufficient tumor penetration in human studies, necessitating alternative molecular designs [21]. In PDAC, FDG-PET also shows limited sensitivity due to occasionally low uptake, high background glucose metabolism, and inflammatory changes [37]. FAPI has demonstrated higher uptake values, particularly in the primary tumor, compared with the initial results obtained with EphA2-targeted imaging [38]; however, these findings may evolve with further evaluation.

Overall, it should be noted that the heterogeneity of the patient cohort, five of the seven patients having undergone multiple prior lines of therapy, may have influenced EphA2 expression patterns. In the present cohort, pretreated patients predominantly exhibited progressive disease. Previous studies have suggested that increased EphA2 expression may be associated with treatment resistance [39], which could theoretically result in higher tracer uptake in this patient subgroup. However, based on the current results, no definitive conclusions can be drawn regarding this relationship. From a methodological perspective, the evaluation of treatment-naïve patients may be particularly informative, as it would allow assessment of EphA2 expression without potential confounding effects of prior therapies and may provide a clearer understanding of baseline EphA2 biology.

A major advantage of [^68^Ga]Ga-BCY18469 was its low physiological uptake in the liver (SUVmean 0.9 ± 0.3), especially when compared with [^18^F]FDG (SUVmean 3.2 ± 0.8) [40]. This may enable improved visualization of liver metastases. This observation is consistent with experience gained using FAPI, as both physiological liver uptake (SUVmean 0.86 ± 0.33) and uptake in distant metastases (SUVmax 7.34 ± 2.48) are comparable to those observed with [^68^Ga]Ga-BCY18469 [38, 41].

Interestingly, some metastases showed no relevant uptake of [^68^Ga]Ga-BCY18469. Several factors may account for this observation, including biological differences such as prior therapy, adaptation of tumor cells to the organ-specific microenvironment or genetic heterogeneity of metastatic lesions compared with the primary tumor [42, 43]. In addition, the large cystic component of the respective liver metastases may further explain low tracer uptake. Lung metastases may also be influenced by respiratory motion artifacts due to their location in the basal right lower lobe.

Beyond these initial findings, three clinically relevant applications of EphA2-targeted PET imaging may be envisioned that future studies should address. First, the early detection in equivocal pancreatic lesions of patients with elevated tumor markers, unspecific symptoms, or inconclusive conventional imaging - particularly in isodense/isointense PDAC where CT and MRI frequently fail to depict the primary mass - [^68^Ga]Ga-BCY18469 PET could support earlier tumor identification and thereby facilitate timely surgical intervention while the disease remains localized [44]. Secondly, improved staging and surgical decision-making in borderline-resectable disease. Here, precisely distinguishing isolated local disease from early occult metastatic spread remains a major clinical challenge. EphA2-PET imaging may allow more accurate definition of resectability and prevent futile surgery in patients with initial radiologically unrecognized distant metastases [13]. Thirdly, patient stratification for EphA2-directed therapy. Because EphA2 overexpression is linked to aggressive tumor biology and metastatic progression, the tracer may serve as a companion diagnostic to identify patients eligible for emerging EphA2-directed therapeutics, including bicycle-toxin conjugates and potentially radioligand therapy concepts based on the same peptide scaffold.

This initial evaluation has several limitations, including the small cohort size, the predominantly pretreated patient population, its retrospective design and the lack of histological confirmation for metastatic disease. In addition, the sometimes considerable interval between EphA2-PET/CT and the corresponding CT/MRI examinations further limits the conclusions that can be drawn regarding diagnostic performance. Moreover, the confirmation of PET-positive-only lesions remains challenging; consequently, false-positive findings cannot be excluded and may arise from inflammatory changes or other benign processes, which is a known limitation even for targeted radiotracers. The absence of histopathological EphA2 expression data prevents the correlation of tracer uptake with receptor density. Furthermore, negative results in EphA2-PET/CT may reflect the heterogeneity of EphA2 expression even in PDAC [9]. This emphasizes the importance of patient selection for EphA2-targeted therapeutic approaches.

## Conclusion

In conclusion, this first clinical translation of EphA2-targeted PET imaging with [^68^Ga]Ga-BCY18469 demonstrates feasibility in visualizing primary and metastatic lesions in patients with PDAC while providing essential biodistribution and dosimetry data. These initial results strongly support the potential of [^68^Ga]Ga-BCY18469 as a diagnostic tool for EphA2-positive malignancies, potentially facilitating personalized treatment strategies. Further studies are warranted to elucidate the clinical role of this radiotracer.

## Supplementary Information

Below is the link to the electronic supplementary material.


Supplementary Material 1 (DOCX 17.3 KB)


## Data Availability

Data available on reasonable request from the corresponding author.
