# Supplementary material for: First-in-human EphA2-targeting PET imaging with the bicyclic radiotracer [68Ga]Ga-BCY18469 in pancreatic cancer patients: biodistribution, dosimetry and initial findings
Source: Eur J Nucl Med Mol Imaging. 2026 Jun 10;53(11):6363–74. doi: 10.1007/s00259-026-07985-1 (PMC13525963; doi:10.1007/s00259-026-07985-1)
Supplement: Supplementary file 1 [file 259_2026_7985_MOESM1_ESM.docx]

Supplementary table 1. Absorbed dose (mGy/MBq) estimates, effective dose following ICRP Publication 103 (*31*).

| **Target organ** | **Mean** | **SD** | **%CV** |
| --- | --- | --- | --- |
| Brain | 1.12E-03 | 1.70E-04 | 15 |
| Breast | 5.96E-03 | 1.31E-03 | 22 |
| Colon wall | 8.79E-03 | 1.13E-03 | 13 |
| Eye lenses | 2.29E-03 | 1.01E-03 | 44 |
| Kidneys | 3.09E-01 | 2.30E-02 | 7 |
| Liver | 1.37E-02 | 6.40E-04 | 5 |
| Lung | 4.34E-03 | 5.87E-04 | 14 |
| Muscle | 7.07E-03 | 1.29E-03 | 18 |
| Oesophagus | 2.20E-03 | 2.09E-04 | 9 |
| Ovaries | 1.81E-02 | 1.31E-02 | 72 |
| Pancreas | 2.44E-02 | 3.99E-03 | 16 |
| Recto-sigmoid colon wall | 2.38E-02 | 6.73E-03 | 28 |
| Red (active) bone marrow | 6.38E-03 | 8.67E-04 | 14 |
| Salivary glands | 1.13E-01 | 8.04E-02 | 71 |
| Skin | 2.42E-03 | 1.05E-04 | 4 |
| Small intestine wall | 1.50E-02 | 2.55E-03 | 17 |
| Spleen | 2.01E-02 | 4.49E-03 | 22 |
| Stomach wall | 1.42E-02 | 2.83E-03 | 20 |
| Testes | 5.87E-03 | n/a | n/a |
| Thyroid | 1.05E-02 | 4.80E-03 | 46 |
| Urinary bladder wall | 1.06E-01 | 2.39E-02 | 23 |
| Effective dose [mSv/MBq] | 1.69E-02 | 2.06E-03 | 12 |

*Breast and ovaries absorbed dose values were calculated for 3 patients. Testes absorbed dose was calculated for 1 patient. n/a: not applicable due to insufficient data*
